# An immune indicator based on BTK and DPEP2 identifies hot and cold tumors and clinical treatment outcomes in lung adenocarcinoma

**DOI:** 10.1038/s41598-023-32276-2

**Published:** 2023-03-29

**Authors:** Tao Han, Yafeng Liu, Jing Wu, Ying Bai, Jiawei Zhou, Chunxiao Hu, Wenting Zhang, Jianqiang Guo, Qingsen Wang, Dong Hu

**Affiliations:** 1 School of Medicine, Anhui University of Science and Technology, Huainan, 232001 People’s Republic of China; 2 Anhui Province Engineering Laboratory of Occupational Health and Safety, Anhui University of Science and Technology, Huainan, 232001 People’s Republic of China; 3 Key Laboratory of Industrial Dust Deep Reduction and Occupational Health and Safety of Anhui Higher Education Institutes, Anhui University of Science and Technology, Huainan, 232001 People’s Republic of China

**Keywords:** Cancer, Computational biology and bioinformatics, Oncology

## Abstract

In lung adenocarcinoma (LUAD), immune heterogeneity of hot and cold tumors has been recognized as one of the major factors affecting immunotherapy and other common treatments. However, there is still a lack of biomarkers that can effectively identify the immunophenotype of cold and hot tumors. First, the immune signatures were obtained based on literature mining, including macrophage/monocyte, IFN-γ response, TGF-β response, IL12 response, lymphocyte activation, and ECM/Dve/immune response. Subsequently, LUAD patients were further clustered into different immune phenotypes based on these immune signatures. Next, the key genes related to the immune phenotypes were screened by WGCNA analysis, univariate analysis, and lasso-cox analysis, and the risk signature was established via the key genes. In additional, we compared the clinicopathological characteristics, drug sensitivity, the abundance of immune infiltration, and the efficacy of immunotherapy and commonly used therapies between patients in the high- and low-risk groups in LUAD. LUAD patients were divided into immune hot phenotype and immune cold phenotype groups. The clinical presentation showed that patients with the immune hot phenotype had higher immunoactivity (including higher MHC, CYT, immune, stromal, ESTIMATE scores, higher abundance of immune cell infiltration, higher abundance of TIL, and enrichment of immune-enriched subtypes) and better survival outcomes than those with the immune cold phenotype. Subsequently, WGCNA analysis, univariate analysis, and lasso-cox analysis identified the genes highly associated with the immune phenotype: BTK and DPEP2. The risk signature, consisting of BTK and DPEP2, is highly correlated with the immune phenotype. High-risk scores were enriched in patients with immune cold phenotype and low-risk scores were enriched in patients with immune hot phenotype. Compared to the high-risk group, the low-risk group had better clinical performance, higher drug sensitivity, and a higher degree of immunoactivity, as well as better efficacy in receiving immunotherapy and common adjuvant therapy. This study developed an immune indicator consisting of BTK and DPEP2 based on the heterogeneity of hot and cold Immunophenotypes of the tumor microenvironment. This indicator has good efficacy in predicting prognosis and assessing the efficacy of immunotherapy, chemotherapy, and radiotherapy. It has the potential to facilitate personalized and precise treatment of LUAD in the future.

## Introduction

Lung cancer is the leading cause of cancer-related morbidity and mortality^1^. Among them, lung adenocarcinoma (LUAD) is the most common lung cancer subtype, accounting for 40% of lung cancers^2^. Despite the increasing number and sophistication of therapeutic approaches for tumors in recent years, the 5-year survival rate of LUAD patients remains only 10–20%^3^. Currently, chemotherapy and molecular targeted therapy are still two common treatment strategies for LUAD, with platinum-based combination chemotherapy being the first-line treatment for advanced LUAD. However, the intra-tumoral heterogeneity of LUAD makes these conventional therapies often fail to achieve the desired results^4^. Therefore, we still need further improvements in the treatment of LUAD.

In recent years, immunotherapy has become increasingly important in the field of oncology treatment^5^, and the therapy has provided clinical benefits in many cancer types such as lung cancer, acute lymphoblastic leukemia, and melanoma^6–8^, yet there are still many patients who cannot benefit from immunotherapy^9^. Current biomarkers for predicting patient response to immunotherapy include tumor mutational burden (TMB)^8^, programmed cell death ligand 1 (PD-L1) expression^10^, and degree of cytotoxic T-cell infiltration^11^. These biomarkers have different accuracy and utility rates, and the robust biomarker of immunotherapy response remains a key challenge in the field^12^.

Previous studies have demonstrated that the efficacy of clinical anticancer therapy could be impacted by various factors in the tumor immune microenvironment (TIME)^13^. Tumor-associated macrophages (TAM), a major component of TIME, can promote tumor growth, angiogenesis, and metastasis^14,15^. The infiltration and activation of T lymphocytes can promote anti-tumor immune responses and inhibit tumor development^16^. Cytokines can act as cancer suppressors or promoters by positively or negatively regulating immune cell functions. Among them, IFN-γ can promote the antitumor immune response by activating T cells^17^. IL-12 promotes the proliferation of natural killer cells and T cells and the production of IFN-γ, thus inducing cellular immunity^18^. In turn, the secretion of TGF-β can suppress antitumor immunity by limiting T-cell infiltration^19^. In addition, the extracellular matrix (ECM) in TIME can also affect tumor-adaptive immune responses by blocking the antigenic expression of antigen-presenting cells (APC) and inhibiting T-cell activity^20,21^. These results illustrate the critical role of complex signaling within the tumor immune microenvironment in clinical treatment^13^. Therefore, exploring the heterogeneity of the tumor immune microenvironment in LUAD may help identify potential biomarkers associated with cancer progression and treatment selection.

In this study, we obtained six immune expression signatures by reviewing the literature and classified patients into two immune phenotypes based on these immune signatures. Subsequently, two key genes, BTK and DPEP2, were selected using WGCNA and LASSO analysis, and an risk signature was established that was highly correlated with the immune phenotypes. The risk signature was significantly correlated with immune infiltration, immunotherapy, and other common treatments, which could be an immune indicator to clinical precision treatment options in the future.

## Materials and methods

### Data collection and download

RNA sequencing data and clinical information of lung adenocarcinoma patients were downloaded from the Cancer Genome Atlas database (TCGA, https://www.cancer.gov/about-nci/organization/ccg/research/structural-genomics/tcga) on the UCSC website at the University of California, Santa Cruz (http://xena.ucsc.edu)^22^. The RNA sequencing data included 517 tumor samples and 59 normal tissue samples (Supplementary Table 1). RNA sequencing data and corresponding clinical information of datasets GSE37745^23^, GSE72094^24^, GSE68465^25^, and GSE126044^26^ were downloaded from the Gene Expression Omnibus (GEO) database (https://www.ncbi.nlm.nih.gov/geo).

### Consensus clustering analysis

The immune enrichment score was calculated based on six immune signatures in LUAD patient tumor samples via single-sample gene set enrichment analysis (ssGSEA)^27^. Immune signatures included "macrophage/monocyte"^28^, "IFN-γ response", "TGF-β response", "IL12 response"^29^, "lymphocyte activation" and "ECM/Dve/immune response (a mix of ECM, muscle/myeloid development and inflammatory response genes)"^30^. Subsequently, TCGA LUAD cancer samples were clustered into different phenotypes via Consensus clustering analysis based on immune enrichment score. Intra-group consistency indicated the optimal number of clusters is k = 2 as the number of clusters (k) increased from 2 to 9. The ssGSEA and cluster analysis were implemented by the R packages "GSVA" and "Consensus Cluster Plus" respectively. The immune enrichment score of these six immune signatures was applied to each LUAD patient by the UMAP algorithm, and the immune subtypes identified by the clustering analysis were integrated to visualize the tumor samples in two dimensions using UMAP1 and UMAP2. UMAP analysis was constructed by the R package "Umap".

### Evaluation of antitumor immunoactivity

The evaluation of the immunological activity characteristics of both groups including 【1】 histopathological slide (H&E staining), the H&E images of LUAD patients from The Cancer Imaging Archive (https://www.cancerimagingarchive.net), and tumor-infiltrating lymphocyte (TIL) patterns assessed via a convolutional neural network^31^. 【2】 TIMER, which is used to assess the abundance of tumor-infiltrating immune cells^32^. 【3】 Immune, stromal and ESTIMATE score, which was used to assess immunoactivity ,was calculated by ESTIMATE algorithm^33^. 【4】 MHC score: Based on the average gene expression in the "core" MHC-I (including HLA-A, HLA-B HLA-C, TAP1, TAP2, NLRC5, PSMB9, PSMB8, and B2M), which indicates the antigen presentation required for T cell recognition of tumor and subsequent T cell-mediated killing^34^. 【5】 CYT score, which reflects the cytolytic activity of immune cells used to kill tumor cells and is calculated as the geometric mean of the genes GZMA and PRF1^35^. 【6】 Tumor microenvironment (TME) subtypes: which include immune-enriched, non-fibrotic (IE); fibrotic (F); immune-enriched, fibrotic (IE/F); immune-depleted (D)^36^.

### Construction of co-expressed gene modules

The LUAD tumor and normal groups were analyzed for differences with a |log_2_FC|≥ 1.5 and *p* < 0.05 criterion to obtain DEGs. The R package "WGCNA" was used to analyze the most relevant modules of DEGs and the core genes in the modules for immunophenotyping. The adjacency matrix is transformed into a topological overlap matrix (TOM), and genes are classified into different gene modules through the TOM-based similarity metric. The optimal soft threshold was 3 when the correlation coefficient was Greater than 0.85, According to the average-linkage hierarchical clustering and the optimal soft threshold power while merging modules with distances less than 0.25, and a minimum module size is 30 to identify key modules. The protein–protein interaction network was calculated through the STRING database (https://string-db.org) and visualized by Cytoscape.

### Construction of the risk signature

First, 19 prognosis-related genes were filtered out from the co-expressed module genes by univariate Cox analysis, followed by a least absolute shrinkage and selection operator (LASSO) analysis to reduce the size of prognosis genes previously filtered by the "glmnet" R package. The Lambda value was 0.043. Each patient’s risk score was calculated as risk score = −0.077*BTK–0.021*DPEP2.

### Drug sensitivity analysis

The "pRRophetic" R package was performed to calculate the half maximal inhibitory concentration (IC_50_) of the drugs for each LUAD patient sample^37^. Then, the IC_50_ of drug was compared between patients in different risk groups.

### Correlation of the risk signature and immunotherapy response

Protein expression of PD-L1 was analyzed by reverse phase protein array (RPPA) analysis and downloaded from The Cancer Proteome Atlas (TCPA, http://tcpaportal.org). Tumor immune dysfunction and exclusion (TIDE) is an approach to predicting immunotherapy responses by pretreatment tumor profiles. The TIDE algorithm is based on two mechanisms of tumor immune evasion: induction of T cell dysfunction in tumors with high cytotoxic T lymphocyte (CTL) infiltration and exclusion of T cell infiltration in tumors with low CTL levels^38^. TIDE score and immune responses of TCGA lung adenocarcinoma patients were calculated by the TIDE website (http://tide.dfci.harvard.edu) after uploading scaled transcriptome profiles. Immunophenoscore (IPS), tumor mutational burden, neoantigen number, clonal neoantigen number, and subclonal neoantigen number of LUAD patients were obtained from the Cancer Immunome Atlas (TCIA, https://tcia.at)^39^.

### UALCAN, TIMER, and TISCH databases

The UALCAN online analysis website (http://ualcan.path.uab.edu/index.html) was used to assess the change in protein levels of BTK and DPEP2 expression^40^. The TIMER database is a publicly available database for analysis of TCGA (https://cistrome.shinyapps.io/timer) about immune infiltration of cancers^32^. The Tumor Immune Single Cell Center (TISCH) is a single-cell RNA sequencing (scRNA-seq) database focused on the tumor microenvironment (TME). The TISCH database could provide detailed cell type annotation at the single cell level to analyze the tumor microenvironment in different cancers^41^ (http://tisch.comp-genomics.org/home).

### Statistical analysis

The significance of differences between the two groups of patients was calculated by the Wilcoxon rank sum test. Univariate Cox analysis and LASSO analysis were applied to identify the key genes in the module for the risk model. Kaplan–Meier(K–M) survival analysis was used to assess the survival differences between the high-risk and low-risk groups. Independent prognostic factors were identified by univariate and multivariate Cox analysis. Categorical data were analyzed by Chi-Squared Test. *p* < 0.05 was considered statistically significant. ns: non-significantly different; *** *p* < 0.001; ** *p* < 0.01; * *p* < 0.05.

## Results

### Construction and validation of the cold and hot immunophenotypes in lung adenocarcinoma

**Figure 1:**
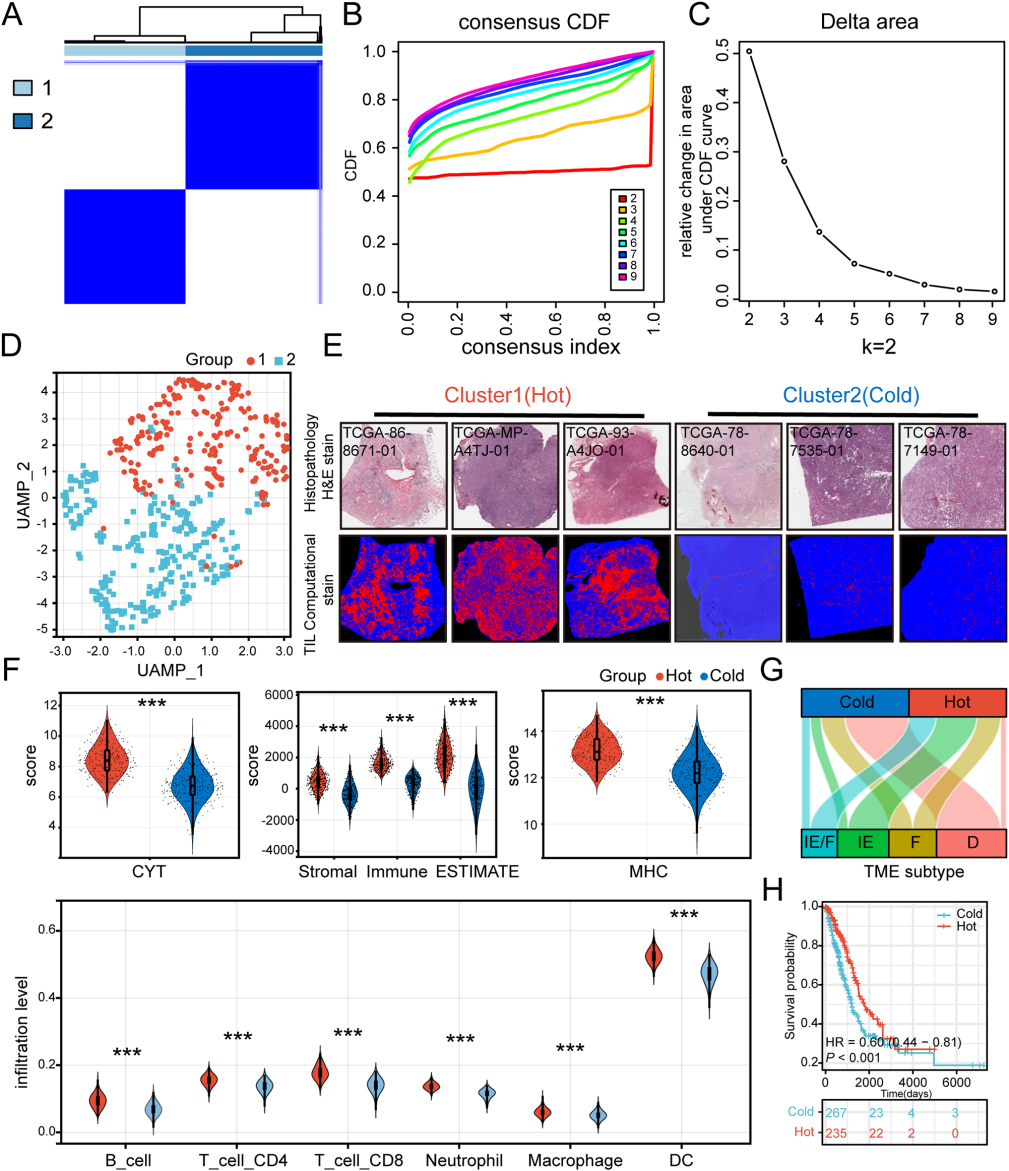
Construction and validation of cold and hot immune subtypes in TCGA-LUAD patients. (**A**–**C**) K = 2 was determined as the optimal value for Consensus clustering analysis. (**D**) UAMP plot of LUAD patients: cluster 1 (hot, n = 243) patients in red and cluster 2 (cold, n = 274) patients in blue. (**E**) Histopathological picture of the tumor tissue (H&E staining) and its TIL pattern (https://cancerimagingarchive.net/datascope/TCGA_TilMap). The TIL pattern is identified by a convolutional neural network, where red pixels denote TIL patches, blue pixels denote non-TIL tissue patches, and black pixels denote non-tissue patches. (**F**) Distribution of CYT score, Immune score, Stromal score, ESTIMATE score, and MHC score of patients in both immune subtypes. The abundance of immune cell infiltration in patients with both immune phenotypes was calculated using TIMER. (**G**) Sankey diagrams of TME subtypes corresponding to different immune phenotypes patients. (**H**) Prognostic differences between patients with different immune phenotypes. (***p < 0.001; **p < 0.01; *p < 0.05).

We used the immune expression signatures ("macrophage/monocyte"^28^, "TGF-β response," "IFN-γ response", "IL12 response"^29^, "lymphocyte activation" and "ECM/Dve/immune" response^30^) to comprehensively depict the immunoactivity of LUAD tumors. The immune enrichment score for these immune signatures was calculated using ssGESA^27^ (Supplementary Table 2), then LUAD patients were divided into 2 groups by consensus clustering analysis (Fig. 1A–C). In addition, the UMAP visualizes both immune subtype populations (Fig. 1D).

In order to investigate the differences in immunoactivity between these two immune populations, we chose Histopathological slides of tumor tissue (H&E staining) and corresponding tumor-infiltrating lymphocytes (TIL) to assess the differences in the level of immune infiltration between these two immune subtype populations. The result demonstrated that patients of cluster 1 have a higher level of immune infiltration compared to patients of cluster 2 (Fig. 1E). Therefore, here we defined Cluster 1 patients as immune hot phenotype and Cluster 2 patients as immune cold phenotype. Later, to further compare the differences in immunoactivity between these two subtypes, we evaluated the anti-tumor immune activity score, including CYT score^35^, immune score, stromal score, ESTIMATE score^33^, and MHC score^34^ in both groups (Fig. 1F). The results suggested that these immune scores were significantly higher in patients of the immune hot phenotype than in patients of the immune cold phenotype. Also, the TIMER algorithm showed a more abundance of immune cell infiltration in the immune hot phenotype group^32^ (Fig. 1F).

We next compared the immunophenotypes with the four well-defined tumor microenvironment (TME) subtypes including immune-enriched, non-fibrotic (IE), fibrotic (F), immune-enriched, fibrotic (IE/F), and immune-depleted(D)^36^. The results showed that most patients with TME subtype (D) were included in the immune cold phenotype group, while patients with TME subtype (IE/F), (IE) were more often included in the immune hot phenotype group (Fig. 1G). These results suggested a strong association between our immunophenotypes and different TME subtypes. Moreover, the Kaplan–Meier (K–M) survival analysis also showed a better prognosis for the immune hot phenotype (Fig. 1H).

### Identification of immunophenotype-associated co-expressed gene modules

**Figure 2:**
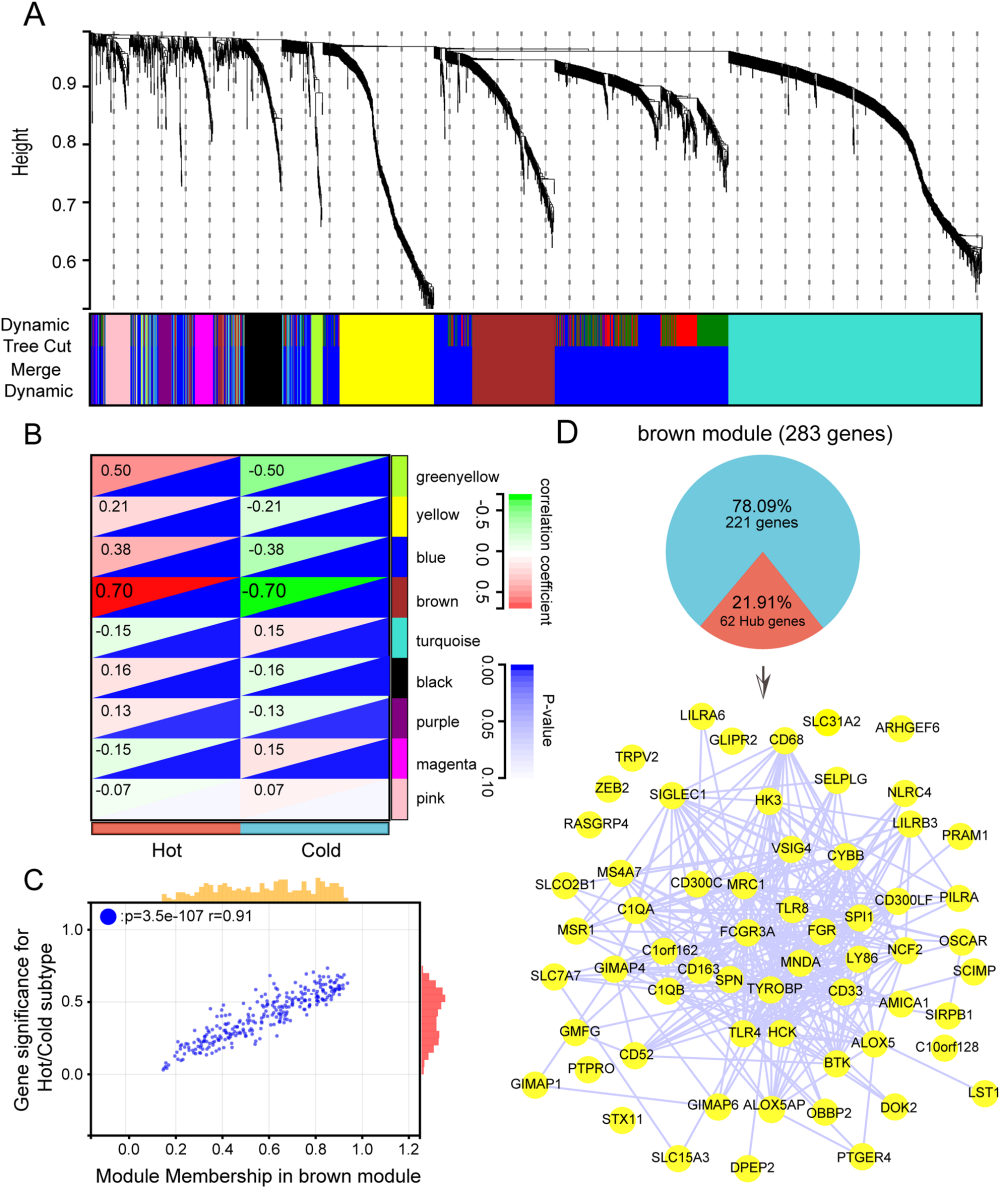
The identification of immunophenotype-related co-expression genes. (**A**) Construction of differential expression gene co-expression modules. (**B**) Correlation analysis of different modules with immune phenotypes. (**C**) Scatter plot of module membership (MM) versus gene significance (GS) in brown module. (**D**) The pie chart demonstrated the proportion of hub genes in brown modules, then the STRING database analyzed the 62 hub genes,61 hub genes were identified and visualized by Cytoscape. (The hub gene: GGTA1 cannot be identified by the STRING database).

In order to find out key gene modules associated with immune phenotypes, we first performed differential gene expression analysis on tumor tissues and corresponding para-cancer tissues from lung adenocarcinoma patients (Supplementary Fig. 1A) and screened out those differential genes with |log2FC|> 1.5, *p* < 0.05 (n = 2537). Then, the total of 2537 genes were assigned to the 9 modules via WGCNA analysis (Supplementary Fig. 1B, C, Fig. 2A). Based on the Person correlation coefficients between the modules and the sample characteristics of each module, it can be concluded that the brown module are closely associated with the hot immune phenotype. The correlation coefficient reached 0.7 (Fig. 2B, *p* value < 0.001). The Module Membership (MM) and Gene Significance (GS) scores were strongly positively correlated with each other in the brown module (Fig. 2C). Then the hub genes were selected in the brown module based on the threshold MM > 0.8 and GS > 0.6, and finally 62 co-expressed hub genes associated with the immune phenotype were obtained. We imported these hub genes into the STRING database and visualized them as protein–protein interactions (PPI) networks by Cytoscape (Fig. 2D).

### Construction of a prognostic risk signature associated with immunophenotypes

**Figure 3:**
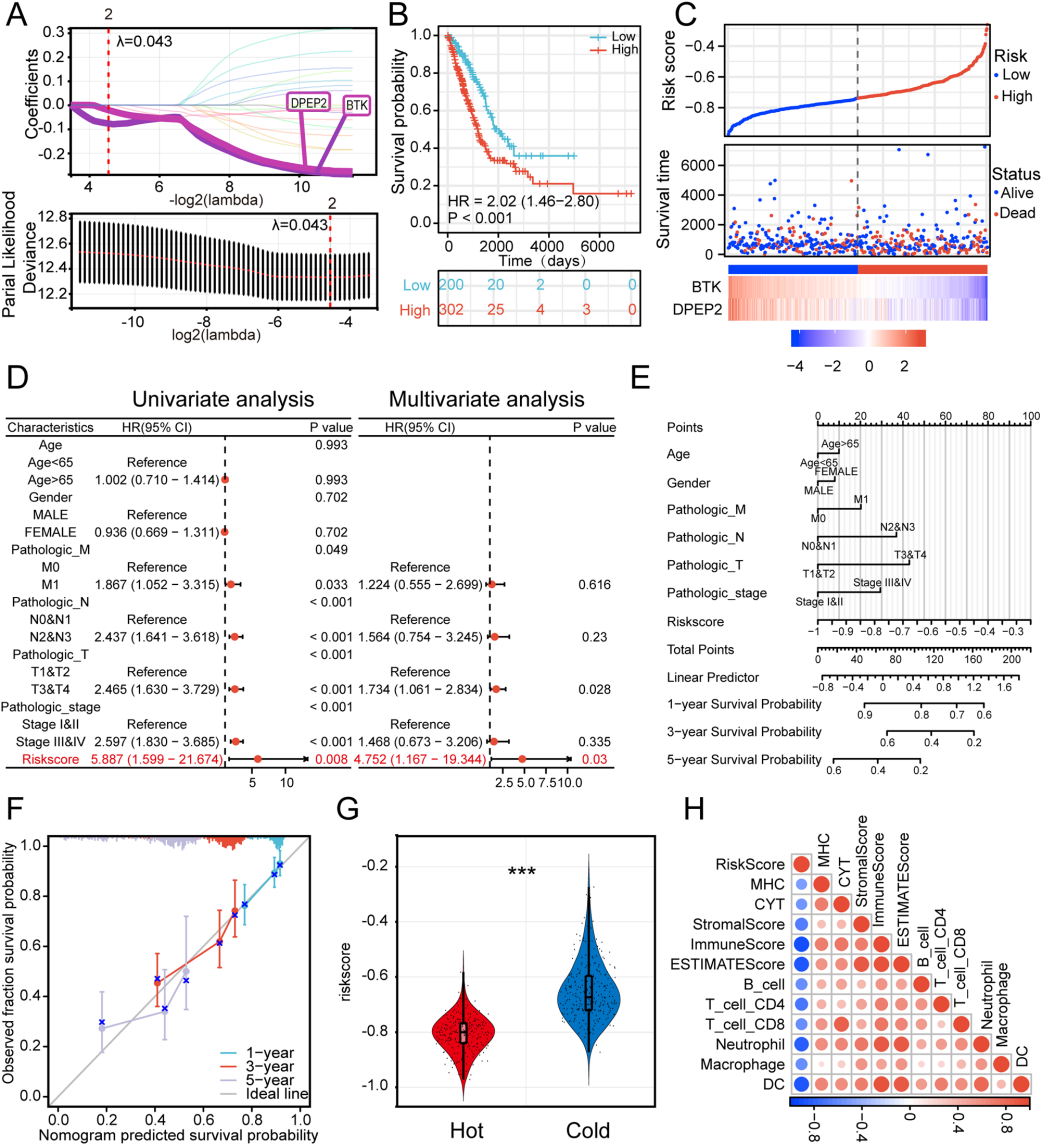
The risk signature is associated with immunophenotypes (**A**) Screened out BTK and DPEP2 and constructed risk model by LASSO analysis with optimal lambda values (**B**) K-M survival analysis of patients in high- and low-risk groups. (**C**) The LUAD patients’ risk score distribution and survival status distribution. (**D**) Univariate and multivariate Cox regression analyses of the association between the prognosis of patients and different clinical-pathological factors(After exclusion of missing values, a total of 336 patients were included in the analysis). (**E**) Construction of a nomogram. (**F**) The construction of the calibration curve for the nomogram model, including three colored lines (blue, red, and purple) represent the performance of the nomogram. A closer fit to the diagonal gray line indicates a better estimation. (**G**) Distribution of risk score in two immune subtypes of LUAD patients. (**H**) Correlation of risk score with patients' immune status using different immune scoring approaches to assess risk score (***p < 0.001; **p < 0.01; *p < 0.05).

The hub genes obtained from the weighted gene co-expression network analysis (WGCNA) brown co-expression module were subjected to univariate Cox analysis, and 19 genes associated with the prognosis were screened out (Supplementary Fig. 2). We set the Lambda value to 0.043, and finally screened for 2 genes: BTK and DPEP2. The risk model equation was: risk score = −0.077*BTK–0.021*DPEP2 (Fig. 3A). K–M survival analysis demonstrated that patients’ survival in the high-risk group was significantly lower than those in the low-risk group: HR = 2.02, 95% CI (1.46–2.80), *p* < 0.001 (Fig. 3B,C).

Univariate Cox regression analysis risk score was statistically associated with OS (HR = 5.887, *p* = 0.008) (Fig. 3D). Considering that the role of other clinical characteristics on prognosis may affect the predictive efficacy of risk score, we further assessed the independent predictive power of risk score using multivariate Cox analysis, which remained an independent prognostic indication of OS in LUAD patients after adjusting for other confounders (HR = 4.752, *p* = 0.03) (Fig. 3D). Next, we integrated the clinicopathological characteristics of the patients with the risk score and constructed a nomogram. The risk score contributed risk points from 0 to 100 in the nomogram (Fig. 3E). The C index of the nomogram was: 0.676, 95% CI (0.649–0.704). And the calibration chart showed a comparative agreement between predicted and observed 1-year, 3-year, and 5-year probabilities of OS (Fig. 3F). These results implied that our model is accurate in predicting patient prognosis.

Then we calculated the correlation between risk signature and immunophenotype. Compared with the immune hot phenotype group, the risk score was significantly higher in our immune cold phenotype group (Fig. 3G). The correlation heat map showed that risk score was significantly negatively correlated with MHC score, CYT score, and ESTIMATE score. TIMER also showed a negative correlation between risk score and the abundance of infiltration of immune cells such as T lymphocytes, B cells, macrophages, DC, and neutrophils (Fig. 3H) (*p* < 0.05, Spearman correlation test). These results demonstrated that the risk signature have promising efficacy in predicting the prognosis and immune status of LUAD patients.

### Validation of risk signature for predicting immunoactivity and prognosis

**Figure 4:**
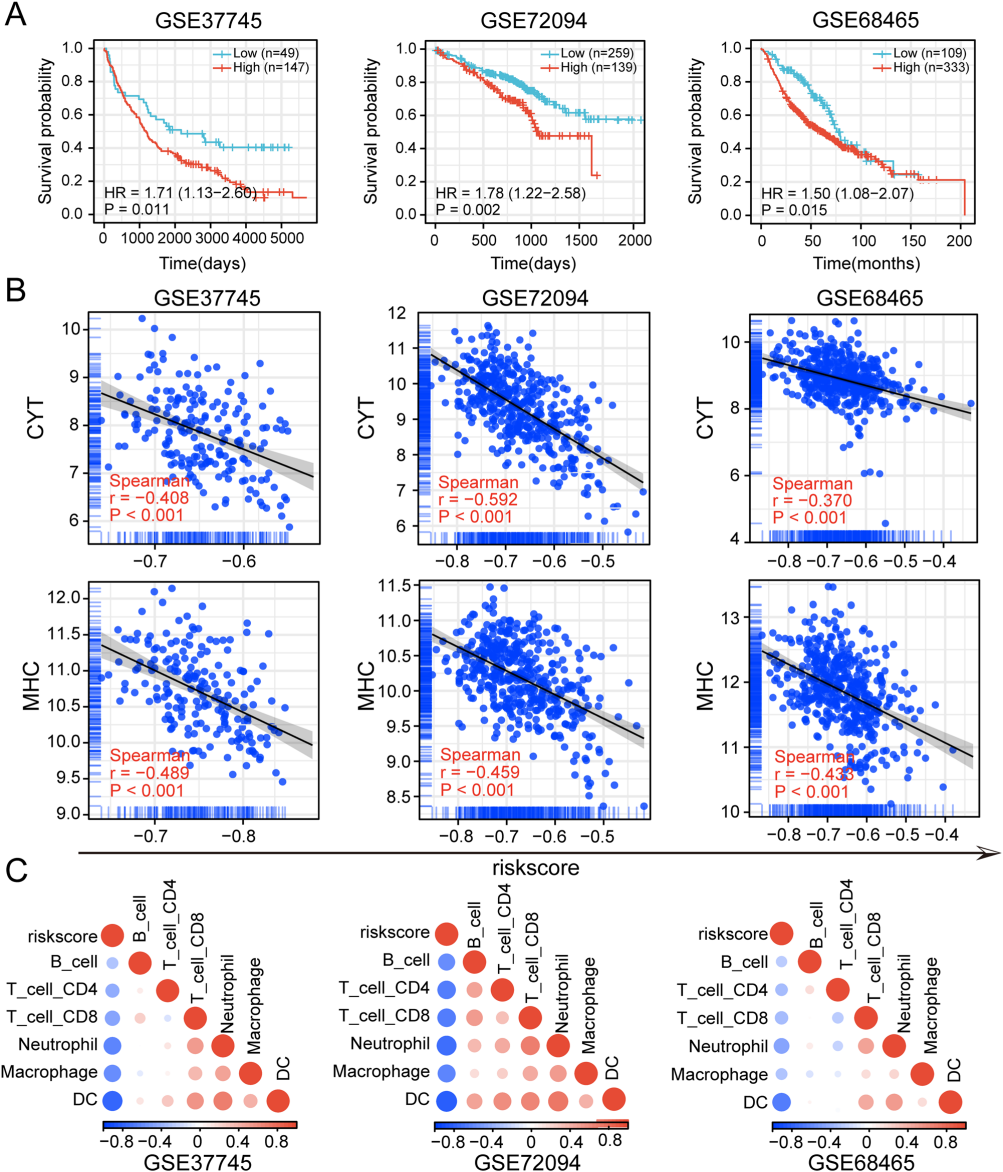
Validation of the risk signature in GEO datasets. (**A**) Correlation of risk scores with prognosis in GEO datasets. (**B**) Correlation of risk scores with CYT score, MHC score in the GEO datasets. (**C**) Correlation of risk scores with immune cell infiltration abundance in the GEO datasets.

To further assess the robustness of the risk signature, two LUAD datasets GSE72094^24^, GSE68465^25^, and one non-small cell cancer (NSCLC) dataset GSE37745^23^ were downloaded from the GEO database to evaluate the model. K-M survival analysis showed that, in these 3 independent datasets, patients with low-risk scores all had a significantly better prognosis than patients with high-risk (*p* < 0.05) (Fig. 4A). Risk scores were also significantly negatively correlated with MHC scores and CYT scores (*p* < 0.001, Spearman's correlation test) (Fig. 4B). TIMER consistently showed a significant negative correlation between risk scores and immune cell infiltration levels in all three validation sets (*p* < 0.01, Spearman's correlation test) (Fig. 4C).

In addition, the ESTIMATE algorithm also showed a negative correlation between risk score and immune score, stromal score and ESTIMATE score, and a positive correlation with tumor purity in the 3 datasets (*p* < 0.001, Spearman correlation test) (Supplementary Fig. 3A–C). Taken together, all these results demonstrated the robustness of the risk signature in predicting patient prognosis and immune status.

### Differences in the distribution and function of risk scores in LUAD patients with different clinical characteristics attributes

**Figure 5:**
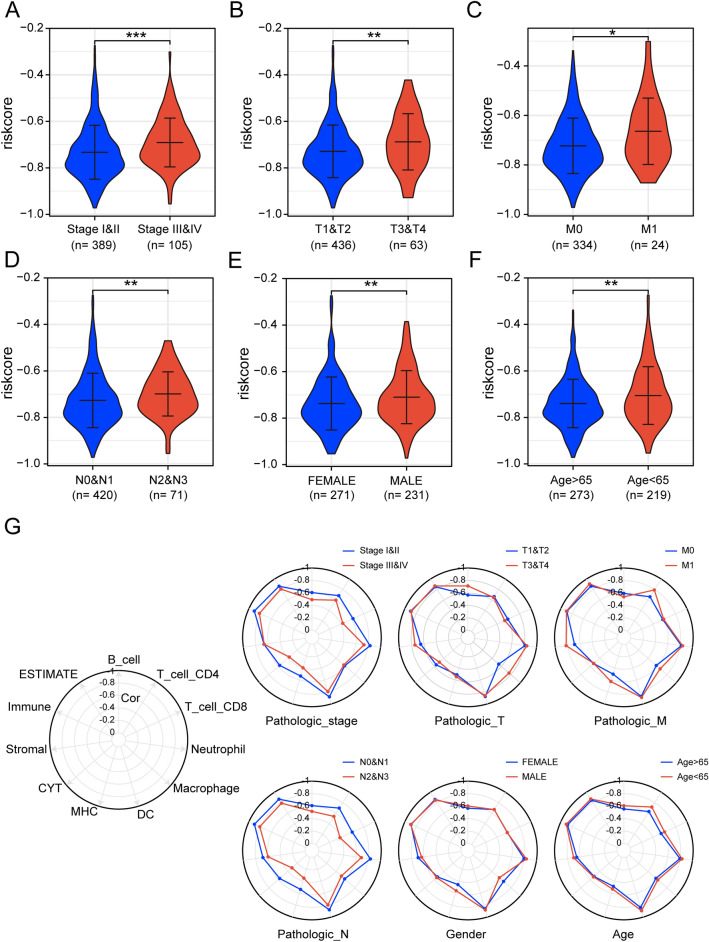
Differences in risk scores in patients with different clinical features. (**A**–**F**) Distribution of risk scores in patients with LUAD at different clinical features. (**G**) Radar plot showing the correlation of risk scores with immune activity in patients with different clinical characteristic attributes, with the innermost circle correlation coefficient being 0 and the outermost circle correlation coefficient being -1. (***p < 0.001; **p < 0.01; *p < 0.05).

Analysis of clinicopathologic characteristics showed that patients with high-risk scores have a more advanced pathological stage (Fig. 5A–D). The results demonstrated that risk scores were strongly related to the clinical features and malignant phenotype of lung adenocarcinoma, which partially explains the poor prognostic clinical outcome in the high-risk group. In addition, Risk scores were higher in male patients than in female patients and higher in patients younger than 65 years of age than in those older than 65 years of age (Fig. 5E,F). We then further explored whether the risk score remained associated with immune infiltration and OS in patients with different clinical features attributes. The results showed that the risk score showed a negative correlation with immune infiltration under all clinical characteristics attributes. Notably, the correlation between risk score and immune infiltration slight decreased in patients with higher pathological stage and N-stage (Fig. 5G). Prognostically, the risk score showed a predictive power for OS at all clinical characteristics (Supplementary Fig. 4A–L).

### The risk signature is associated with immunotherapy prognosis in lung adenocarcinoma

**Figure 6:**
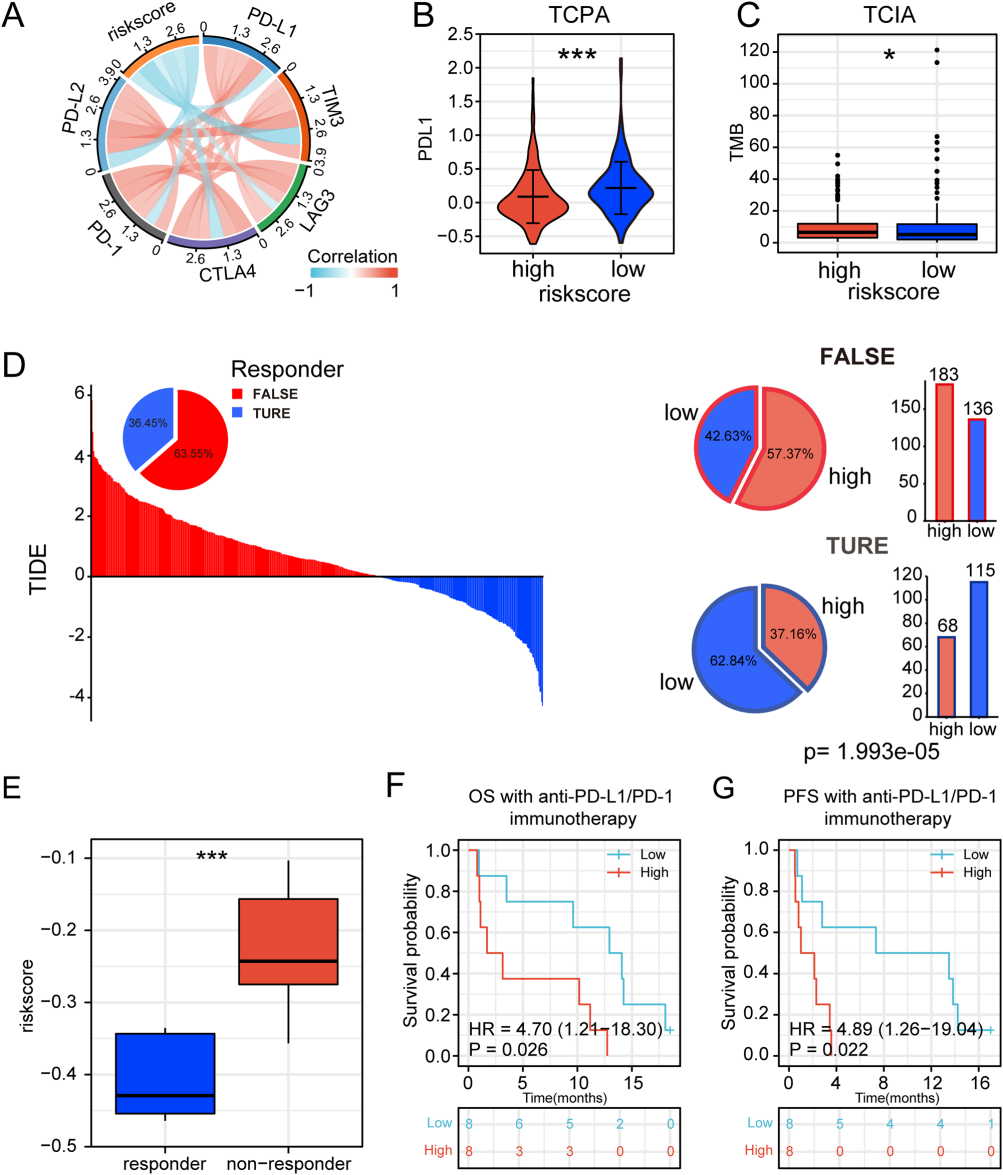
Relationship between risk signature and immunotherapy. (**A**) Correlation between risk scores and immune checkpoints. (**B**) Distribution of PD-L1 protein levels in high-risk and low-risk populations based on the TCPA dataset. (**C**) Distribution of TMB in high- and low-risk populations based on TCIA. (**D**) TIDE scores and response outcomes of immunotherapy in LUAD patients. Distribution of risk scores among TIDE predicted immunotherapy response or non-response groups, chi-square. *p* < 0.001. (**E**) Distribution of risk scores among immunotherapy responders and non-responders. (**F**, **G**) Association of risk score with OS and PFS in patients receiving immunotherapy (ns: no significant difference. (***p < 0.001; **p < 0.01; *p < 0.05).

Predictive markers of immune response play a vital role in the course of immunotherapy in patients. Therefore, we explored the relationship between risk scores and several common immune checkpoints. The results showed that PD-1, PD-L1, PD-L2, CTLA4, LAG3, and TIM3 were negatively correlated with the risk score (Fig. 6A). Similarly, risk scores were negatively correlated with these immune checkpoints in the 3 validation sets (Supplementary Fig. 5A–C). Compared to the high-risk group, the low-risk group of LUAD patients from the TCPA database had higher levels of PD-L1 protein (Fig. 6B). These results suggested that the risk score may be closely related to the formation of a dysfunctional TIME. We next compared TMB, number of neoantigens (including number of clonal neoantigens and number of sub-clonal neoantigens), and IPS score between high-risk and low-risk groups of LUAD patients from TCIA^39^. The results suggested that high-risk patients had higher TMB and neoantigens (Fig. 6C, Supplementary Fig. 5D). The low-risk group had higher IPS-PD1/PD-L1/PD-L2, IPS-CTLA4, and IPS-PD1/PD-L1/PD-L2 + CTLA4 scores, but there was no significant difference in IPS scores (Supplementary Fig. 5E).

Then we introduced tumor immune dysfunction and exclusion (TIDE)^38^, and the results showed that the low-risk group (45.81%, 115/251) were more likely to respond to immunotherapy than those in the high-risk score group (27.09%, 68/251) among the patients predicted by TIDE to be likely to respond to immunotherapy (chi-square, *p* < 0.001, Fig. 6D). Considering the association between risk scores and clinicopathological features, we further analyzed the predictive efficacy of risk scores for immunotherapy in patients with different features. The results showed that the risk score had stable predictive efficacy for immunotherapy response in LUAD patients with different clinical features (Supplementary Fig. 5F).TIDE scores were lower in the low-risk group than in the high-risk group, implying that the low-risk group benefited more from immunotherapy compared to the high-risk group (Supplementary Fig. 5G). In addition, the T-cell exclusion score was significantly lower in the low-risk group than in the high-risk group (Supplementary Fig. 5H), but the T-cell dysfunction score was higher than in the low-risk group (Supplementary Fig. 5I).

To further validate the predictive effect of risk score on immunotherapy response, we introduced an immunotherapy cohort GSE126044^21^ (n = 16). The results demonstrated that the risk score was significantly higher among non-responders compared to responders (Fig. 6E). The area under the ROC curve (AUC) for the risk score was 0.927 when differentiating between responders and non-responders (Supplementary Fig. 5J). Furthermore, OS and PFS survival analysis also showed that patients in the low-risk group had longer overall survival time and progression-free survival time than the high-risk group (Fig. 6F,G). In conclusion, the above results demonstrated the effectiveness of risk scores in predicting the efficacy of immunotherapy.

### The risk signature is associated with the efficacy of common clinical treatments

**Figure 7:**
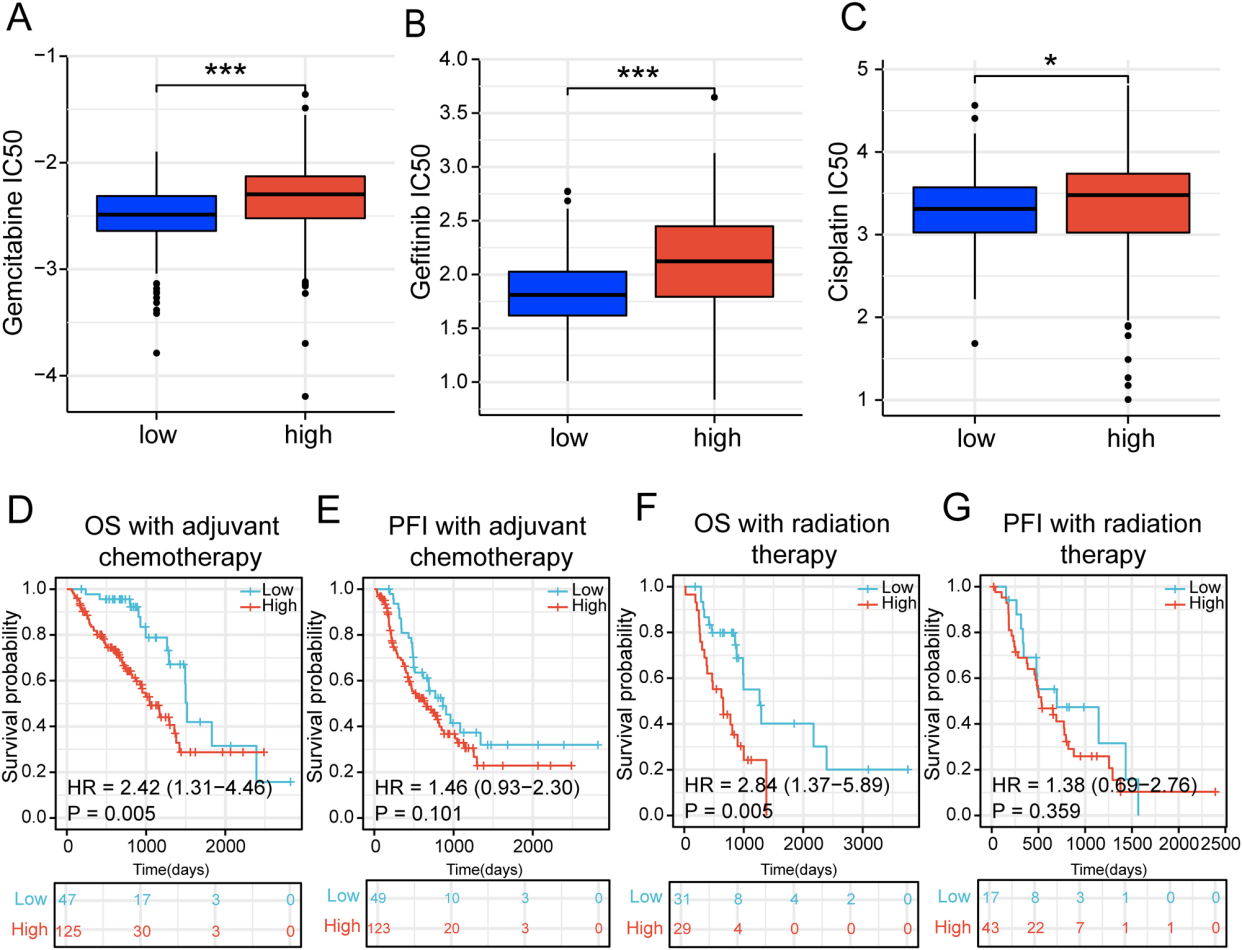
Association of risk signature with common clinical treatments. (**A**–**C**) Distribution of drug IC_50_ concentrations in patients in the high-risk group and low-risk group. (**D**, **E**) Survival curves of OS (overall survival) and PFI (progression-free interval) in TCGA-LUAD patients receiving chemotherapy. (**F**, **G**) Survival curves of OS and PFI in TCGA-LUAD patients receiving radiotherapy. (***p < 0.001; **p < 0.01; *p < 0.05).

Tumor immune microenvironment is commonly considered to be related to chemotherapeutic efficacy^42^. Therefore, We investigated the association between risk scores and the sensitivity of common anti-tumor drugs for lung cancer^43,44^. The results showed that the IC_50_ of the drugs (gemcitabine, cisplatin, and gefitinib) was significantly lower in the low-risk group than in the high-risk group (Fig. 7A–C).

Afterward, we compared the risk score to the prognosis of treated LUAD patients. The results showed that higher risk scores tend to imply a worse prognosis in LUAD patients receiving radiotherapy and chemotherapy. chemotherapy (OS: HR = 2.42, *p* = 0.005; PFI: HR = 1.46, *p* = 0.101) (Fig. 7D,E), and radiotherapy (OS: HR = 2.84, *p* = 0.005; PFI: HR = 1.38, *p* = 0.359) (Fig. 7F,G). All of these results suggested a greater benefit from chemotherapy or radiotherapy for LUAD patients in the low-risk group compared to the high-risk group.

### BTK and DPEP2 expressed in macrophages to promote immune infiltration

Considering the robustness of the risk score in predicting prognosis and immune activity in lung adenocarcinoma , we further explored the role of BTK and DPEP2, which are key genes comprising the risk score. It was found that tumor tissues had lower expression of BTK and DPEP2 at mRNA and protein levels compared to normal tissues (Supplementary Fig. 6A,B). In addition, patients with high BTK and DPEP2 expression tended to have longer survival (Supplementary Fig. 6C). In terms of clinical features, the expression of BTK and DPEP2 was lower in higher pathological stages (Supplementary Fig. 6D,E). These results suggest that BTK and DPEP2 may be involved in the antitumor process of lung adenocarcinoma.

**Figure 8:**
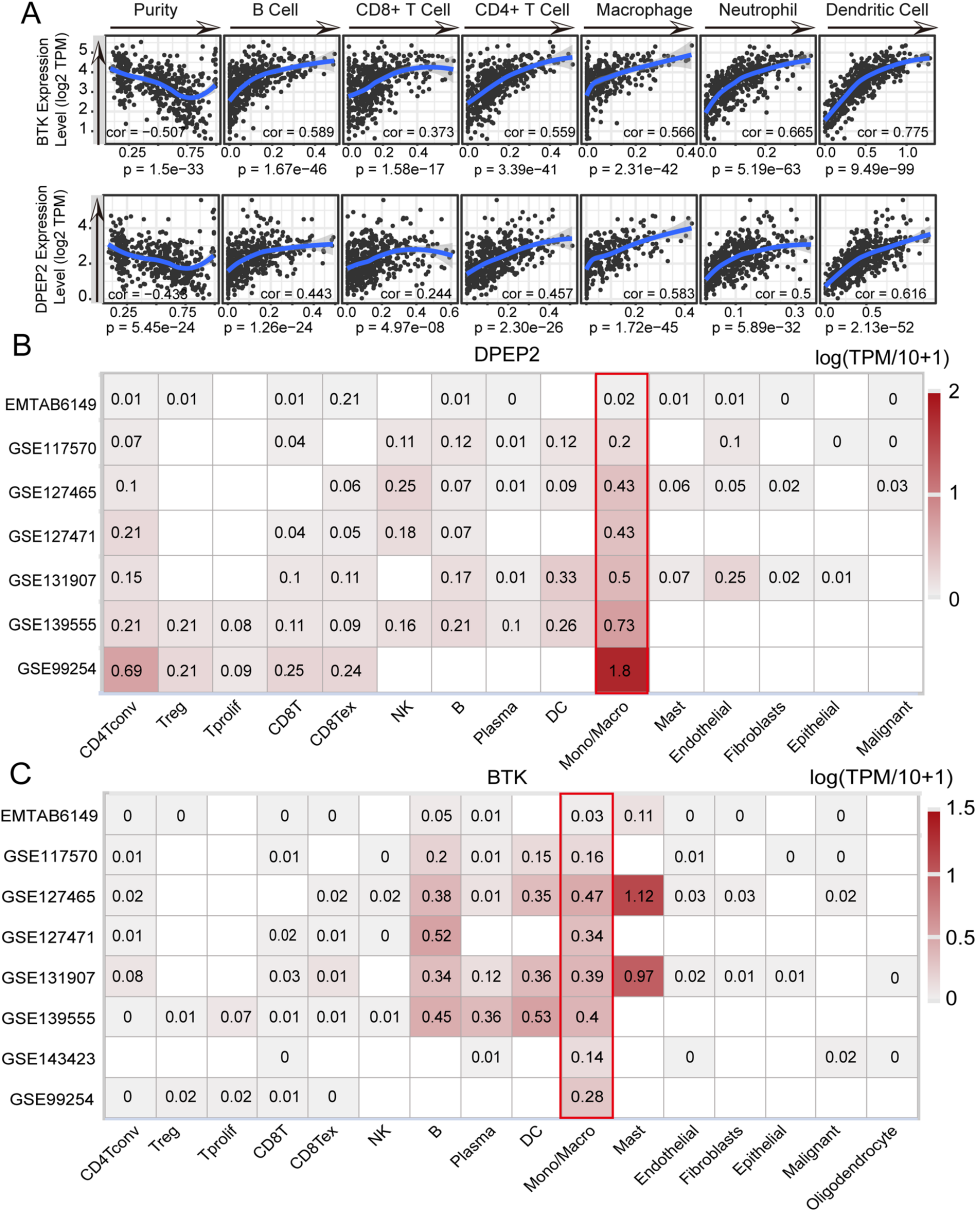
Association of BTK and DPEP2 with immune infiltration. (**A**)TIMER database to calculate the correlation of BTK and DPEP2 with immune cell infiltration in LUAD patients. (**B**, **C**) Calculation of BTK and DPEP2 expression levels in different cell subpopulations using the TISCH website.

To further investigate the role of BTK and DPEP2 in the remodeling of the tumor immune microenvironment. We next explored the link between BTK and DPEP2 and immune cell infiltration. As expected, the TIMER database showed a significant positive correlation between BTK and DPEP2 expression and immune cell infiltration and a significant negative correlation with tumor purity (Fig. 8A). Meanwhile, several NSCLC single-cell sequencing datasets from TISCH^41^ showed that both BTK and DPEP2 were mainly expressed on monocytes and macrophages (Fig. 8B,C). The results imply that BTK and DPEP2 may regulate the abundance of immune infiltration and promote anti-tumor immunity by affecting the function of macrophages and monocytes.

## Discussion

A growing number of studies have found that the effectiveness of clinical therapy is often influenced by the tumor immune microenvironment^13,45–47^. Therefore, we need a biomarker that reflects the tumor immune microenvironment to help guide the clinical treatment strategy for LUAD.

In this study, LUAD patients were divided into immune hot and immune cold phenotypes by six immune signatures related to the immune microenvironment. Histopathological sections of tumor tissues, TIL patterns, and assessment of antitumor immune activity showed that patients with the immune hot phenotype had a better immune status. The prognostic analysis also showed a longer survival time for patients in the group with immune hot phenotype.

We identified core gene modules that were important in the immune hot phenotype by WGCNA analysis. Then we screened the core genes via univariate Cox analysis and lasso-cox analysis to construct a risk model which was highly correlated with the immune phenotype. Compared to the low-risk group, patients in the high-risk group were significantly more enriched in the immune cold phenotype group and had lower infiltration abundance of immune cells, immune scores, and shorter survival time. In addition, the risk score remained an independent predictor of OS in LUAD patients after adjusting for other confounding factors. Risk signature also had good efficiency in the validation of the 3 GEO datasets. These results illustrated the good efficiency and robustness of our risk features in identifying the immune status of patients as well as survival prognosis.

Correlation analysis with clinical features showed that high-risk scores were significantly enriched for known malignant clinical features, which is consistent with the poor prognosis of patients with high risk scores. Also, this suggests that risk scores may evaluate tumor progression and malignancy. In addition, we further analyzed the correlation of risk score with immune infiltration and prognosis in different pathologic stages. Interestingly, we found that the correlation between risk score and immune infiltration score decreased in higher pathological stages. Here, we hypothesize that as pathological staging progresses, the expression of BTK and DPEP2, which constitute the risk score, gradually decreases, as does their ability to promote immune infiltration, ultimately, making the correlation between risk score and the abundance of immune infiltration in TME diminish. However, the underlying mechanisms remain to be further explored.

We next explored the relationship between risk score and immunotherapy response in LUAD patients. The results demonstrated that the risk score was negatively correlated with several common immune checkpoints PD-L1, PD-1, LAG3, PD-L2, TIM3, and CTLA-4^48^. Consistently, PD-L1 protein expression was higher in the low-risk group than in the high-risk group. These results suggest that risk scores probably predict the dysfunctional TIME. Immunophenoscore (IPS), as a predictor, can evaluate the immunotherapy response of patients efficiently^39^, and in our study IPS-PD-1/PD-L1/PD-L2, IPS-CTLA4, IPS-CTLA4 + PD1/PD-L1/PD-L2 scores all suggested that patients with LUAD in the low-risk group were more suitable for immune checkpoint inhibitor therapy. In addition, recent studies have shown that genomic instability affects immune response and immunotherapy efficacy^49^, in our study, patients in the high-risk group had a higher frequency of tumor mutation burdens. It was shown that high TMB in patients not receiving immunotherapy tended to imply higher pathological staging and poorer survival outcomes^50^, similar to the clinical characteristics of the patients in the high-risk group of this study. This phenomenon explains to some extent why TMB was more enriched in the high-risk group. Among patients receiving immunotherapy, it is generally believed that patients with high TMB respond better to immunotherapy than those with low TMB^51,52^. Interestingly, a recent study points to a potential central role of the tumor microenvironment including CD8T cells, macrophages, and dendritic cells in TMB predicting immunotherapy^53^. Furthermore, Sinha et al. in their study suggested that different immune activities can influence the predictive efficacy of TMB^54^. Therefore, based on these results, it is reasonable to believe that it is the lower immune infiltration of TME in the high-risk group that leads to the elimination of the immunotherapeutic stratification ability of TMB.

In order to further understand the predictive effect of risk signature on the immune response. We introduced the TIDE algorithm to predict the likelihood of patient response to immunotherapy^38^. The results showed that the low-risk group was more likely to respond to immunotherapy. At the same time, the risk score demonstrated robust predictive efficacy for immunotherapy in patients with different clinical features. The TIDE scores and T-cell exclusion scores were higher in the high-risk group. The higher TIDE scores represent a higher likelihood of immune evasion and poorer treatment outcomes. Therefore, the lower-risk group with lower TIDE scores may have a better prognosis^38^. In the immunotherapy cohort of LUAD, GSE126044^26^, our risk score also showed good efficacy in distinguishing between responders and non-responders to immunotherapy. The non-responders had significantly higher risk scores than the responders. In conclusion, all these results suggest that our risk score can be used to predict immunotherapy response in patients with LUAD.

Analysis of the relationship between risk scores and IC_50_ concentrations of drugs that commonly used to treat lung cancer like cisplatin, gefitinib and gemcitabine^43,44^, were higher in the high-risk group. It implies that patients in the low-risk group may have better efficacy when treated with chemotherapy or targeted therapy. As expected, K-M survival analysis showed significantly better OS in the low-risk group than in the high-risk group in patients receiving chemotherapy. At the same time, the risk score is also efficient in predicting the survival of patients receiving radiotherapy. These results suggest that the risk score can be used to predict the response of LUAD patients after receiving radiotherapy and chemotherapy.

Considering the relationship between the risk model and the presence of immune infiltration, and immunotherapy, we further explored the possible roles of the two genes used to construct the model: BTK and DPEP2 genes in the tumor microenvironment of lung adenocarcinoma. BTK is present a tyrosine kinase in normal B cells at all stages of maturation except for plasma cells, and it is mainly downstream of pre-BCR and BCR. In cancer, the pathogenesis of BTK in mature B-cell malignancies has been well studied^55^, but its role in lung adenocarcinoma is unknown. DPEP2 was originally identified as a membrane-bound dipeptidase that hydrolyzes LTD4 to LTE4, and both substrates and products of DPEP2 have been associated with inflammatory diseases^56,57^. Studies have shown that DPEP2 can regulate inflammation caused by macrophages and is also associated with macrophage differentiation^58,59^. Our study showed that the expression of BTK and DPEP2 in tumor tissues of LUAD patients was significantly and positively correlated with the infiltration of immune cells, the TISCH database showed that BTK and DPEP2 were mainly expressed in monocytes/macrophages^41^. We hypothesize that BTK and DPEP2 genes may alter the immune microenvironment of tumors by affecting the function of macrophages and the anti-tumor therapeutic effect, which deserves further research.

Although the risk signature constructed in this work can be used as an immune indictor to predict immunotherapy response and prognosis of LUAD patients, there are still some flaws existing. First, all cases in this study were retrospective samples, needing to be further validated. Secondly, the ways used to validate the effect of the immune signature score are mainly indirect assessment of the predictive power of the signature score for immunotherapy response, and only a few patients within the immunotherapy cohort were used to validate the predictive effect of the signature score. Therefore, some robust direct evidence is still necessary in the future.

## Conclusions

In conclusion, this study clustered LUAD into hot and cold immunophenotypes by known immune signatures and established an immune indictor consisting of BTK and DPEP2 associated with hot and cold immunophenotypes, which showed good efficiency in identifying hot and cold immune phenotypes and assessing prognosis, clinical radiotherapy and chemotherapy efficacy, immune infiltration, and immunotherapeutic effects in LUAD.

## Supplementary Information

Supplementary Information 1.

Supplementary Information 2.

Supplementary Information 3.

## Data Availability

The datasets analyzed during the current study are available in the [Gene Expression Omnibus] (https://www.ncbi.nlm.nih.gov/geo/), including GSE37745, GSE72094, GSE68465 and GSE126044 datasets.
