# Supplementary material for: An immune indicator based on BTK and DPEP2 identifies hot and cold tumors and clinical treatment outcomes in lung adenocarcinoma
Source: Sci Rep. 2023 Mar 29;13:5153. doi: 10.1038/s41598-023-32276-2 (PMC10060209; doi:10.1038/s41598-023-32276-2)
Supplement: Supplementary file 1 — Supplementary Information 1. [file 41598_2023_32276_MOESM1_ESM.docx]

­
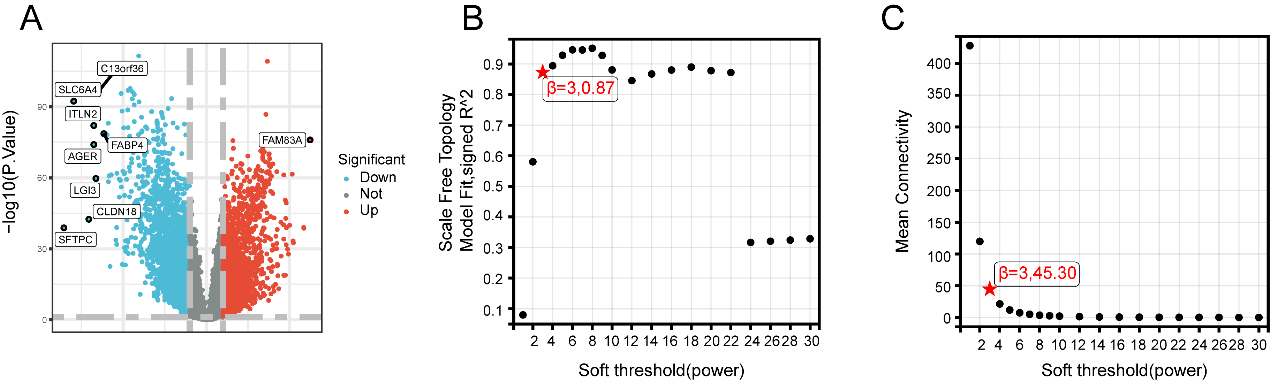


**Supplementary Fig.1** **Screening of differential expression genes and WGCNA analysis.** (A) Volcano map of differential genes between tumor and normal tissues. |log_2_FC| of the tagged genes greater than 6. (B-C) Selection of soft threshold values. The correlation coefficient was greater than 0.85 when the soft threshold value was 3.


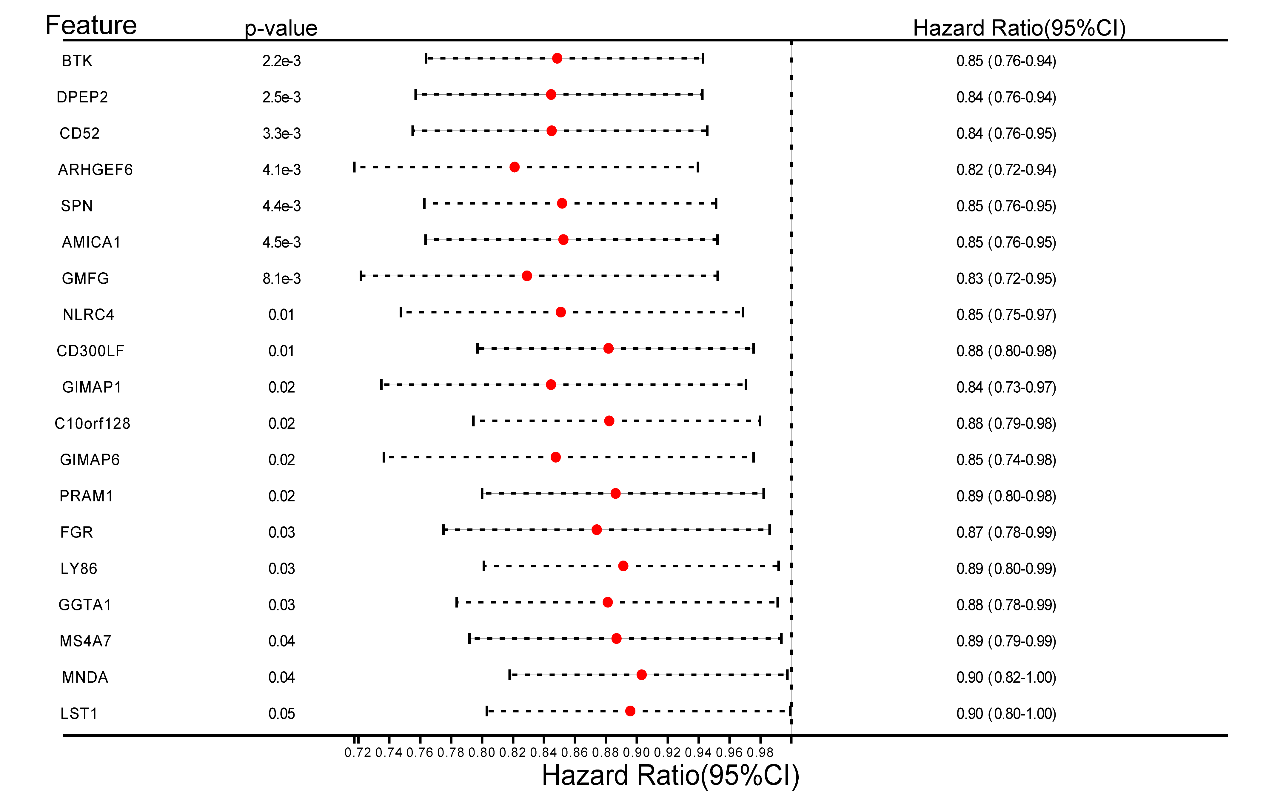


**Supplementary Fig.2** **Univariate cox analysis revealed the significant prognostic effect of 19 immunophenotype-associated hub genes.**


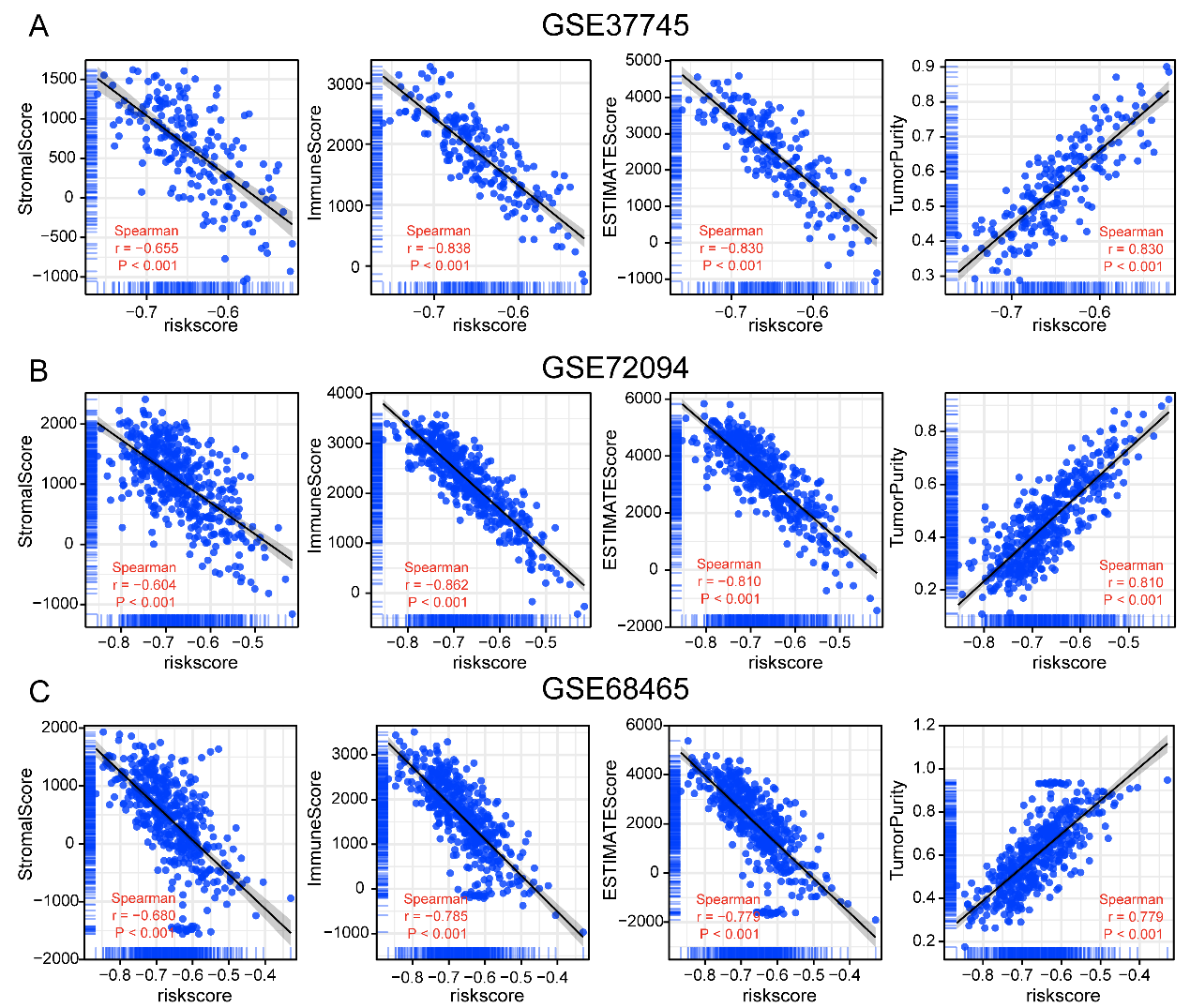


**Supplementary Fig.3 Correlation of risk scores with immune scores.** Correlation of risk score with the stromal score, immune score, ESTIMATE score, and tumor purity for the (A) GSE37745 dataset, (B) GSE72094 dataset, and (C) GSE68465 dataset.


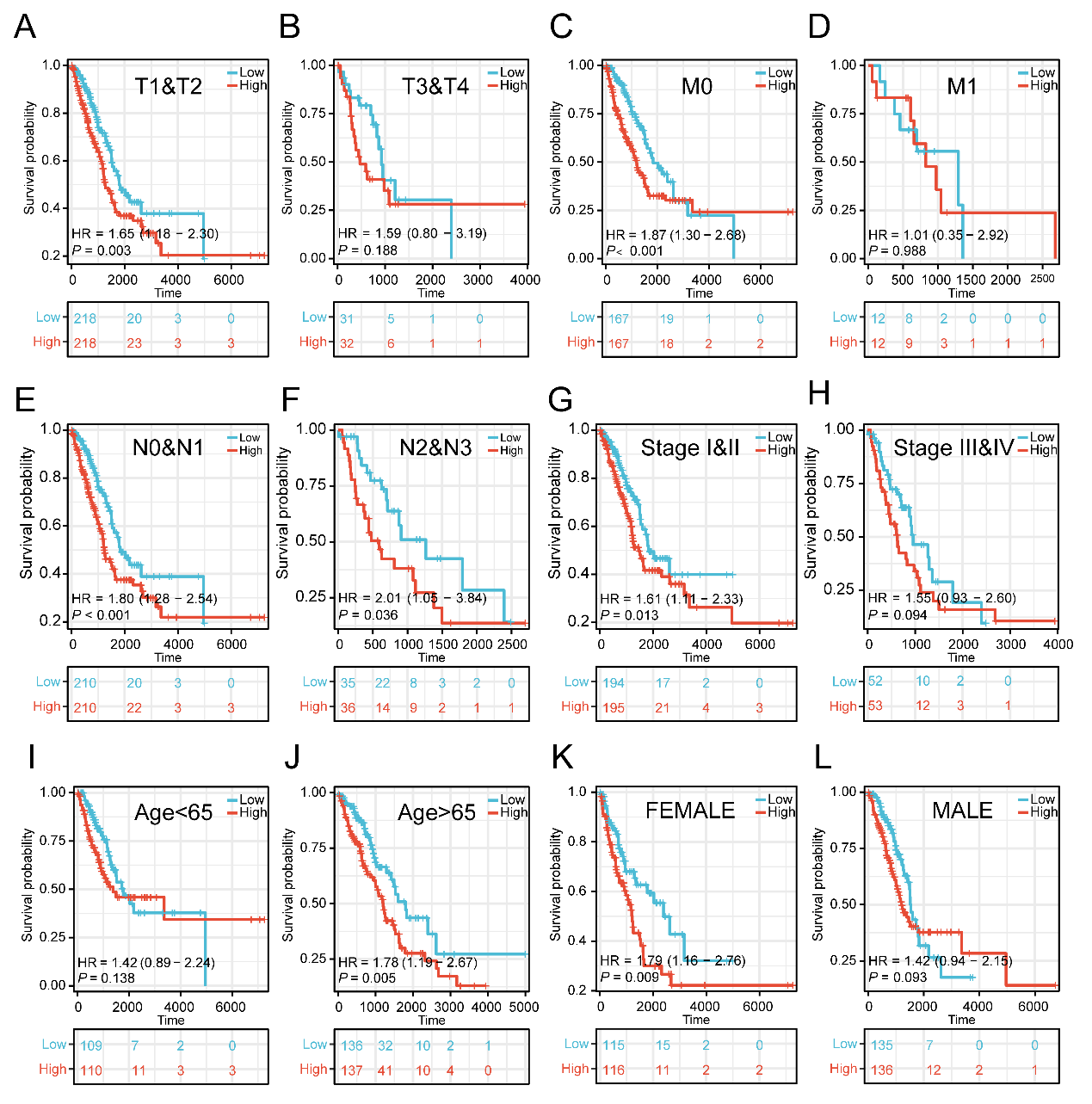


**Supplementary Fig.4 Efficacy of risk scores in predicting OS in different clinicopathological characteristics.** (A-L) K-M survival analysis of risk scores in different clinical characteristics.


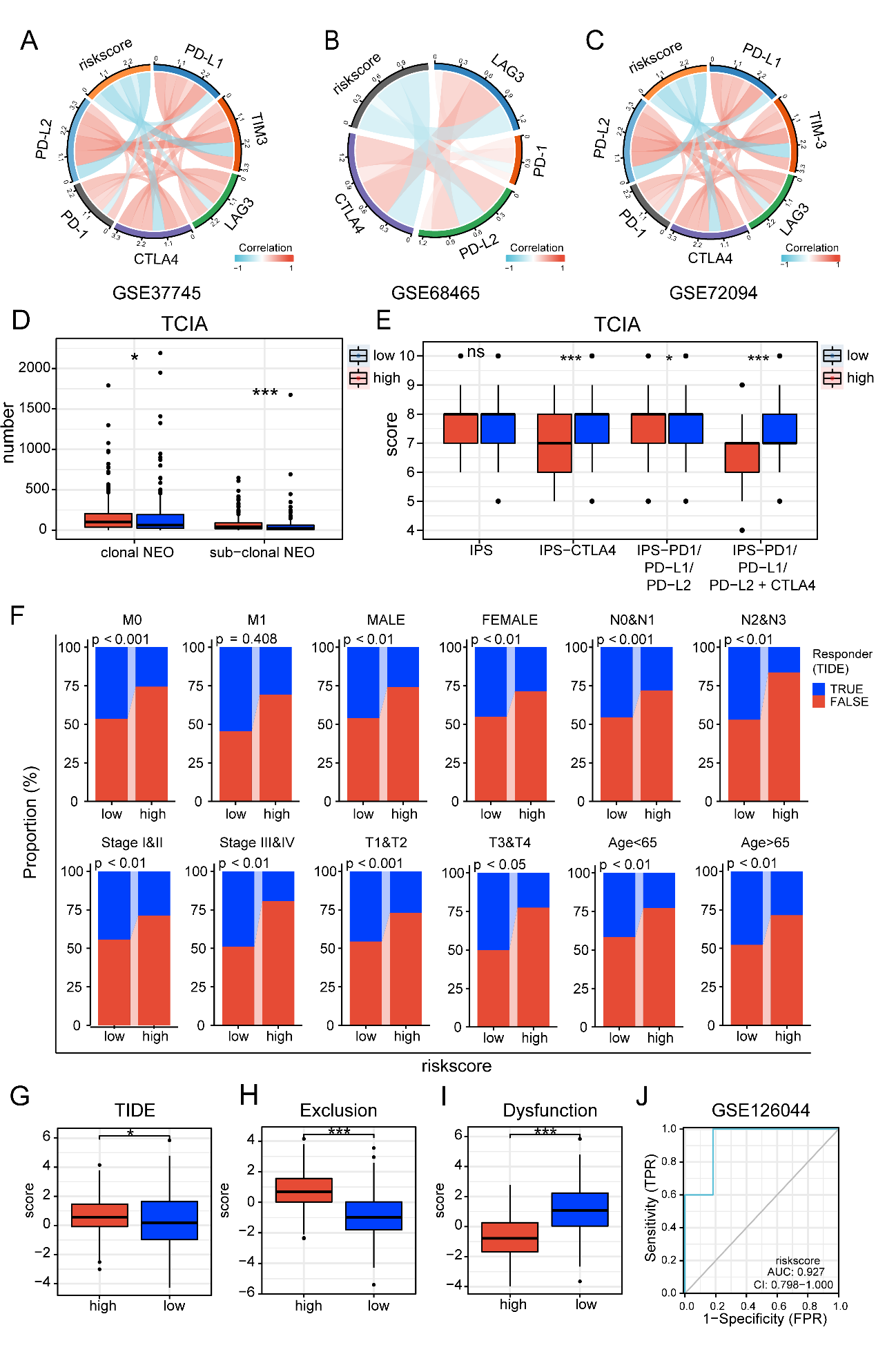


**Supplementary Fig.5 Prediction of risk score on immunotherapy response.** (A-C) Correlation between risk scores and immune checkpoints for the 3 GEO datasets. (GSE68465 is missing the gene expression of PD-L1(CD274) and TIM-3(HAVCR2). (D-E) The distribution of neoantigens, sub-clonal neoantigens, and IPS scores in high- and low-risk populations based on TCIA. (F) Stacked plots showing the proportion of responders and non-responders predicted by the TIDE algorithm in different risk groups for different clinicopathological features. Categorical variables using chi-square test. (G-I) The distribution of TIDE score, Exclusion score, and Dysfunction between high-risk and low-risk. (J) ROC curves of risk score distinguishing responders from non-responders in the immunotherapy cohort. (*** p < 0.001; ** p < 0.01; * p < 0.05)


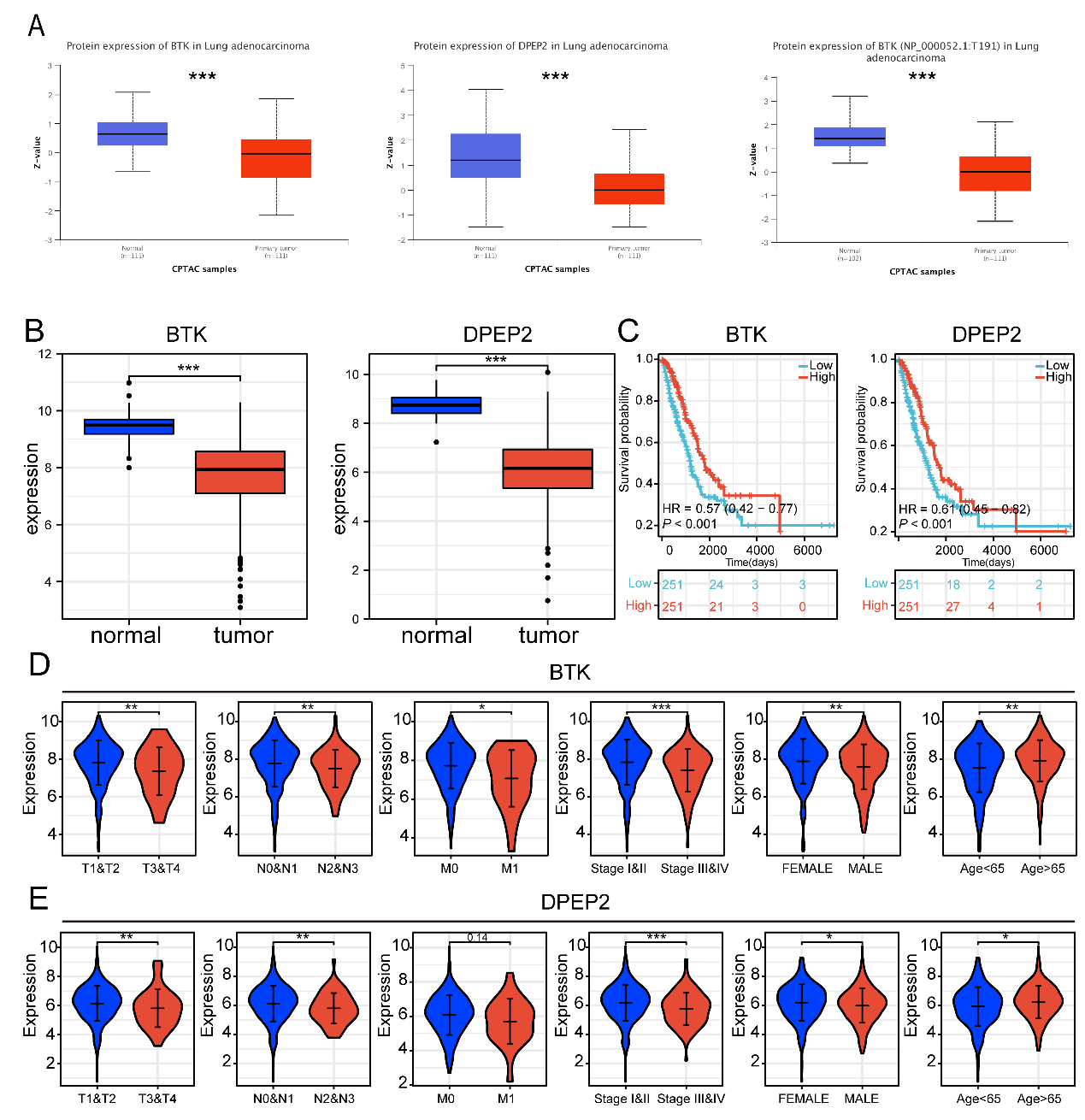


**Supplementary Fig.6 Differential expression of BTK and DPEP2 in lung adenocarcinoma.** (A) Comparison of protein expression levels of BTK and DPEP2 between tumor tissues and normal tissues of LUAD patients using the CPTAC database on the UALCAN website. Differences in phosphorylation levels of BTK between tumor tissues and normal tissues of LUAD patients using the CPTAC database on the UALCAN website. (B) Gene expression differences between BTK and DPEP2 in LUAD tumor tissues and normal tissues. (C) Association of BTK and DPEP2 expression with the prognosis of LUAD patients. Distribution of (D) BTK and (E) DPEP2 in patients with different clinical features. (*** p < 0.001; ** p < 0.01; * p < 0.05)
